# Oxidative Stress in Chronic Kidney Disease

**DOI:** 10.1155/2016/8375186

**Published:** 2016-08-08

**Authors:** Alexandra Scholze, Joachim Jankowski, José Pedraza-Chaverri, Pieter Evenepoel

**Affiliations:** ^1^Department of Nephrology, Odense University Hospital and Institute of Clinical Research, University of Southern Denmark, 5000 Odense C, Denmark; ^2^Institute for Molecular Cardiovascular Research, RWTH Aachen University, University Hospital, 52074 Aachen, Germany; ^3^Faculty of Chemistry, Department of Biology, National Autonomous University of Mexico (UNAM), 04510 Mexico City, Mexico; ^4^Laboratory of Nephrology, Department of Immunology and Microbiology, University of Leuven, 3000 Leuven, Belgium

Chronic kidney disease (CKD) is associated with a high risk for cardiovascular and all-cause mortality and premature aging [1, 2]. Already in 2002, Himmelfarb et al. suggested that oxidative stress is a “unifying concept of cardiovascular disease in uremia” [3]. For several years now, researchers and clinicians in nephrology have been working on the proof and extension of this concept, on refining and arguing its details, and on the desired implementation of respective therapies and biomarkers. The present special issue aims to inform the readership on latest developments on various aspects of oxidative stress research in CKD and to foster further research in the field.

E. Dounousi et al. contributed an interesting study to strengthen the hypothesis that oxidative stress is involved in the pathogenesis of cardiomyopathy in CKD. They investigated the association between the Paraoxonase-1 Q192R gene variant and cardiomyopathy. Paraoxonase-1 (PON1) belongs to the Paraoxonase family and exerts a protective effect against lipoprotein oxidation. PON1 is diminished in patients with CKD and has been suggested as a marker for antioxidant status [4]. In their article now E. Dounousi et al. show that the R allele of the Q192R variant in the PON1 gene is dose-dependently related to the severity of left ventricular hypertrophy and left ventricular dysfunction in CKD. They also proved that the group of patients that was homozygous for the R allele showed significantly higher concentrations of the lipid peroxidation marker 8-isoprostane in plasma. The authors discuss that the patients enrolled in this study were all Caucasians recruited from a restricted geographical area and point out that confirmatory studies in CKD patient cohorts with different geographical and ethnic background are desirable.

K. Poulianiti et al. present a systematic review about systemic redox imbalances in CKD. They focus on the impact of disease severity, anemia therapy, mode of dialysis treatment, and antioxidant interventions in both hemodialysis and peritoneal dialysis patients. Obviously, the imbalance in systemic redox status is evident already at an early stage of CKD and becomes more profound with kidney disease progression. Evidence in early CKD is however limited and needs to be expanded. Also, the authors conclude that hemodialysis therapy per se seems to exert a negative influence on systemic redox status but that other dialysis modalities have not proven so far advantages with respect to the occurrence of oxidative stress. The authors suggest that supplementation with antioxidants might be considered as an early intervention to halt premature cardiovascular disease in CKD. Intervention studies, however, especially also in this patient group with early CKD are hampered by small sample size and relatively short duration.

J. Yu et al. explored the role of cell-targeted antioxidant interventions and more specifically investigated the effects of the cell-permeable superoxide dismutase (SOD) mimetic Mn(III)tetrakis (4-benzoic acid) porphyrin (MnTBAP) on renal oxidative stress and fibrosis in a mouse model of CKD. They found that fibrotic transition and mitochondrial dysfunction after transforming growth factor-*β*1 treatment of mouse tubular epithelial cells could be reduced by pretreatment with MnTBAP. Furthermore, in uremic mice, intraperitoneal injection of MnTBAP resulted in a reduction of renal fibrosis in the remnant kidney. The authors suggest that MnTBAP therapy might serve as a promising strategy to prevent renal fibrosis in CKD via antagonistic effects on mitochondrial-derived oxidative stress and subsequent protection of mitochondrial function. These findings appear promising and necessitate further investigation especially in the light of the recently discussed prooxidative activities of metal porphyrins used as SOD mimetics in biological systems [5].

The reasons that underlie the wish for the application of antioxidant therapy in CKD are numerous including a reduction of CKD-related increased cancer incidence. One strategy to reduce cancer risk is interventions to reduce genomic damage which is also increased in CKD patients. Such approaches necessitate a reliable analysis of DNA. Here, the article by N. Schupp et al. in this issue will be read by nephrological researchers and clinicians likewise with great interest. The authors provide an in depth review of biomarkers for DNA damage in CKD. They explain, compare, and evaluate the most common methods that are currently used to quantify DNA damage in CKD patients, discuss the markers' potential to predict clinical outcomes, and provide information about ongoing efforts for standardization.

The review by J. Pedraza-Chaverri et al. discusses some newer aspects of pathogenesis of oxidative stress and antioxidant therapy in CKD. The authors provide an overview over selected current antioxidant interventions and discuss new, not yet fully elucidated concepts of oxidative stress genesis. So the impact of mitochondrial alterations in CKD and possible connections to the development of cardiovascular disease are highlighted. Also, the authors provide information on two controversial players for the redox balance in CKD: hyperuricemia and vitamin D deficiency. Underlying molecular mechanisms and pharmacological interventions are presented. Finally, emerging therapies for diabetic nephropathy, including sodium-glucose cotransporter 2 inhibitors and alternative, traditional medicines, are discussed.

In summary, this special issue demonstrates that knowledge on causes and consequences of oxidative stress in CKD is rapidly expanding. Many gaps, however, remain, calling for additional research efforts to unravel its pathogenesis and to test therapeutic approaches. No simple antioxidant solutions are at hand and solid clinical outcome data both for biomarker research and for interventions studies are necessary. Moreover, when analyzing oxidative stress related molecular changes and the impact of antioxidant interventions, important variables such as CKD stage, genetic background, current therapies, and comorbidities should be accounted for.
